# Molecular Dissection Using Array Comparative Genomic Hybridization and Clinical Evaluation of An Infertile Male Carrier of An Unbalanced Y;21 Translocation: A Case Report and Review of The Literature 

**DOI:** 10.22074/ijfs.2015.4619

**Published:** 2015-12-23

**Authors:** Alfredo Orrico, Giuseppina Marseglia, Chiara Pescucci, Ambra Cortesi, Paola Piomboni, Andrea Giansanti, Francesca Gerundino, Roberto Ponchietti

**Affiliations:** 1 Molecular Medicine Unit, Azienda Ospedaliera Universitaria Senese, Siena, Italy; 2 Medical Genetics, Misericordia Hospital, Grosseto, Italy; 3 Diagnostic Genetic Unit, Department of Laboratory, Careggi University Hospital, Firenze, Italy; 4 Department of Molecular and Developmental Medicine, University of Siena, Azienda Ospedaliera Universitaria Senese, Siena, Italy; 5 Genitourinary Unit, University of Siena, Azienda Ospedaliera Universitaria Senese, Siena, Italy

**Keywords:** Translocation, Azoospermia, Hypogonadism, Y Chromosome

## Abstract

Chromosomal defects are relatively frequent in infertile men however, translocations between the Y chromosome and autosomes are rare and less than 40 cases of Y-autosome translocation have been reported. In particular, only three individuals has been described with a Y;21 translocation, up to now. We report on an additional case of an infertile man in whom a Y;21 translocation was associated with the deletion of a large part of the Y chromosome long arm. Applying various techniques, including conventional cytogenetic procedures, fluorescence in situ hybridisation (FISH) analysis and array comparative genomic hybridization (array-CGH) studies, we identified a derivative chromosome originating from a fragment of the short arm of the chromosome Y translocated on the short arm of the 21 chromosome. The Y chromosome structural rearrangement resulted in the intactness of the entire short arm, including the sex-determining region Y (SRY) and the short stature homeobox (SHOX) loci, although translocated on the 21 chromosome, and the loss of a large part of the long arm of the Y chromosome, including azoospermia factor-a (AZFa), AZFb, AZFc and Yq heterochromatin regions. This is the first case in which a (Yp;21p) translocation has been ascertained using an array-CGH approach, thus reporting details of such a rearrangement at higher resolution.

## Introduction

Chromosome anomalies contribute frequently to reproductive failure in men, accounting for approximately 7% of all infertility cases and 10-15% of azoospermia (1,3). Sex chromosomal abnormalities, in particular, may be considered relatively common, as the Klinefelter syndrome occurs in approximately 0.1-0.2% in newborn males, and Y chromosome microdeletions in the azoospermia factor (AZF) regions have been detected in approximately 8% of nonobstructive azoospermic and 5% of severely oligozoospermic men (4,5). Some discrepancies between different investigations, probably due to ascertainment biases and/ or real differences in populations studied, do not alter the assumption that variations of the Y chromosome, in particular microdeletions, impact on spermatogenesis. More rarely, rearrangements of the Y chromosome may take the form of translocations in which variable Y-chromosome-derived material translocates to different recipient chromosomes. When unbalanced, such a rearrangement can be responsible for a large spectrum of different clinical phenotypes, depending on the translocation breakpoints on Y chromosome and autosomes and/or on the chromosomal regions possibly deleted (6,10). The resulting phenotypes may range from Turner syndrome to infertile males with azoospermia or, at least, with severe oligozoospermia as cardinal feature (11,12). Occasionally, a Y-autosome translocation may be characterized for an apparent discrepancy between the karyotype and the expressed sexual phenotype. In fact, an apparently 45,X karyotype may be associated with maleness, due to the translocation of the testis-determining gene [sex-determining region Y (SRY)] onto an autosome. Less than 40 cases of Y-autosome translocation have been reported in literature still now; all of them are different from each other as regard to the autosome and/or the involved regions of the Y chromosome (13,20). However, in all the previous reports, the characterization of the critical regions involved in such a chromosomal imbalance was carried out by conventional karyotyping or fluorescence in situ hybridisation (FISH) studies. Here, we describe for the first time the results of a detailed molecular dissection using array comparative genomic hybridization (array-CGH) for the characterization of the chromosomal regions involved in a Y;21 translocation occurred in an infertile man. Applying this molecular cytogenetics method, we were able to accurately map the specific genomic regions involved in the translocation breakpoints, offering refined molecular data in such a condition. 

## Case report

### Clinical findings

An apparently healthy man, 34-year-old, attended our services after a two years history of infertility. An informed consent was obtained from the patient after the aims and procedures of the investigation were fully explained. He appeared as a well-developed male, 170 cm tall, weighing 68 kg. Physical examinations revealed normal male habitus except for a high-pitched voice and testis slightly smaller in size: at scrotal echography, the right testis was evaluated about 15 ml in volume and the left approximately 12 ml. Repeated semen analysis revealed azoospermia. Endocrinological examinations showed hypergonadotrophic hypogonadism, with elevated follicle stimulating hormone (FSH, 24.6 mU/ml), moderately low total serum testosterone level (2.74 ng/ ml), very low free testosterone (3.74 pg/ml), and normal levels of sex-hormone-binding globulin (34.8 nmol/l) and luteinizing hormone (LH, 4.2 mU/ml). The patient, who received preand posttest genetic counselling, refused a testicular biopsy for diagnostic purposes and the possibly recovery of germ cells. 

### Cytogenetic analysis

High-resolution chromosome analysis [Quinacrine (QFQ) banding] was performed from blood lymphocyte cultures according to standard cytogenetic procedures. FISH was carried out according to manufacturer’s instructions, using centromeric probes for chromosomes Y (DYZ3, Yp11.1-q11.1; Kreatech Diagnostics) and 13/21 (D13Z1/D21Z1, 13p11.1-q11.1 and 21p11.1-q11.1; Kreatech Diagnostics), subtelomere-specific probes for both arms of the sex chromosomes (DXYS130, Xp/Yp telomeres; DXYS224, Xq/Yq telomeres; Kreatech Diagnostics) and probes for the whole chromosome Y and 21 (Kreatech Diagnostics). Lymphocyte preparations from male with normal karyotype were used as controls for the FISH assays. 

### Molecular genetic analysis

Array-CGH was performed using the Human Genome CGH 4X180K Microarray Kit (Agilent Technologies, USA), according to the manufacturer’s protocols. The Agilent Feature Extraction software has been used to perform image analysis. In order to correct systematic spatial and intensity biases, the results were normalized using the lowess function. Normalised log2ratio values were calculated and breakpoint identification was performed applying the Shifting Level Model (SLM) segmentation algorithm (21). The probabilistic classification of each segmented region into biologically motivated status (loss, neutral or gain) was performed by FastCall algorithm (22). A rearrangement was defined by the deviation of at least three consecutive probes, with a practical average resolution of about 100 kb. 

**Fig.1:**
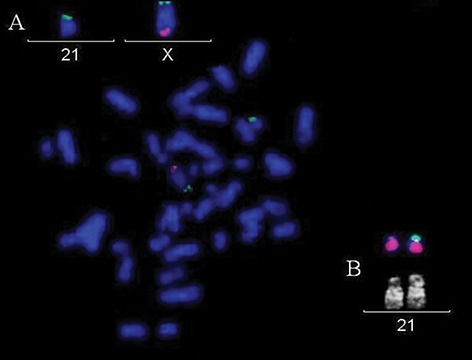
A patient’s metaphase fluorescence in situ hybridization (FISH) image showing a 45,X karyotype due to the loss of chromosome Y: the red probe identifies the Xq and Y q subtelomere regions (marker DXYS224), the green probe identifies Xp and Y p subtelomere regions (marker DXYS130). A. FISH detail of X and 21 cromosomes shows the green signal at the p-telomere of the X chromosome and at the p-telomere of the derivative chromosome 21 The probe for the long arms of the Y and X chromosomes (red signal) shows fluorescence illumination only of the tip of the long arm of the chromosome X (normal X chromosome): the red signal to be referred to Yq is missed and B. Detail of Quinacrine (QFQ) and FISH of the 21 chromosomes. The FISH probe of the Yp (labelled in green) results to be translocated to the distal short arm of the derivative chromosome 21, while the normal chromosome 21 shows only the counterstained 21-chromosome painting probe (labelled in red).

In our patient, the Y chromosome was not detectable at a first conventional chromosome analysis, revealing the appearance of a 45,X0 karyotype. As the result was inconsistent with the phenotype of the patient and some additional material detected on the terminal part of 21p chromosome, might be suspected of Y-derived material, we applied FISH analysis. By this approach, we detected a derivative chromosome originating from a fragment of the short arm of the chromosome Y translocated on the short arm of the 21 chromosome (Fig .1). By applying dual-color FISH using alpha-satellite probes respectively for Y (Yp11.1-q11.1) and 21 (21p11.1- q11.1) chromosomes, we identified two centromeres at the derivative chromosome, originated from centromeres of both Y and 21 chromosomes. 

**Fig.2:**
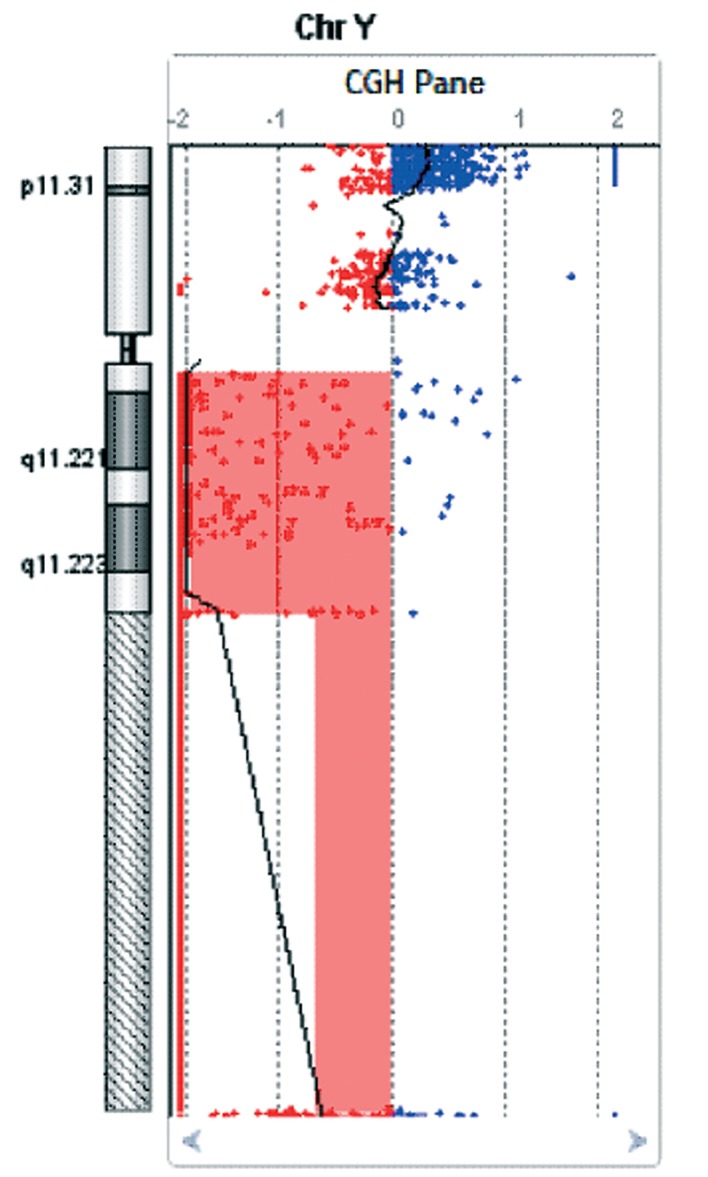
Chromosome Y array comparative genomic hybridization (array-CGH) plot. The area highlighted in red shows the loss of about 45 Mb of the chromosomal region Yq11.21-q12 spanning from 13,992 kb to 59,031 kb (according to UCSC Genome Browser GRCh37/hg19).

As a whole, the derived chromosome could be described by FISH as [45,X,der(21)t(Y;21)(q11;p11). ish der(21)(wcpY+,wcp21+,DXYS130+,DYZ3+, D13Z1/D21Z1+,DXYS224)]. To further characterize the rearrangement, array-CGH analysis was carried out and showed a Yq deletion of about 45 Mb ± 0.2 Mb, spanning from 13,992 kb to 59,031 kb, respectively the first and the last probes on the array platform. According to the University of California Santa Cruz (UCSC) Genome Browser, GRCh37/hg19 deletion breakpoints mapped in Yq11.2-q12 ( arr Yq11.21- q12(13.992.304-59.031.421)x1 (Fig .2). 

## Discussion

Chromosomal imbalances can severely affect male fertility. The prevalence of karyotype abnormalities in infertile males are reported about 8-10 fold higher than in general population (23,24). In particular, reciprocal translocations between autosomes are evaluated to occur in approximately 1% of severely oligoor azoospermic men, versus 0.1% of general population (25). Moreover, one of the most common genetic contributions to male infertility is represented by Y chromosome microdeletions involving the three AZF regions located on the distal Yq11 region. In contrast to such submicroscopic chromosomal imbalances, translocations involving the Y chromosome occur very rarely. To our knowledge, less than 30 cases of material exchange between a Y chromosome and an autosome has been reported in literature, only three of them involving the 21 chromosome (16,26,27). More recently, a mosaicism for an unbalanced Y;21 translocation with the loss of chromosome 21 material has been reported (28). In our case, we report on a constitutional translocation between the short arms of the chromosomes Y and 21 [( 45, X, t(Yp;21p) (p12;p1.1)], with the loss of Yq material. The breakpoints were detected in the proximal region of the long arm of the Y chromosome (Yq11) and in the distal region of the short arm of chromosome 21 (21p12), resulting in a derived chromosome with both Y and 21 centromeres. More specifically, array-CGH showed a Yq11.21q11.2 deletion of about 45 Mb, encompassing a genomic region that includes about 200 genes and transcripts, the majority of which involved in the events of sperm maturation, specifically expressed in the male germline. The Y chromosome structural rearrangement results in a conserved short arm of the Y chromosome, including the SRY and the short stature homeobox (SHOX) loci, although translocated on the 21 chromosome, and the loss of a large part of the long arm of the Y chromosome, including the AZFa, AZFb, AZFc and Yq heterochromatin regions. In agreement with the three cases previously reported (16,26,27), the retention of the SRY gene allowed the complete masculinization of the patient, although azoospermic. Among the secondary sexual traits, only a persistent high-pitched voice resulted as an additional and distinctive phenotypic sign in our patient, supporting the well-known negative relationship between the voice pitch and circulating levels of testosterone in men (29,30). 

Due to the complexity of the information regarding his genetic status, as previously agreed with patient, he was seen in several rounds of genetic consultation after the test, alone and in the company of his partner, to disclose the result and to discuss all the possible implications. As we inform that sperm retrieval from a testicular biopsy is considered ineffective for males with such a Y chromosomal deletion (entire AZF region), a more invasive evaluation was refused and no testicular tissue studies were performed for histological examinations. Overall, the patient did not show adverse psychological reactions after the disclosure of genetic information: he did not exhibit anxiety and/or depression symptoms and refused a proposed psychological support as considering it unnecessary. These findings are consistent with the notion that the psychological distress associated with the communication about positive genetic test results may be reduced by careful pre- and post- test genetic counseling. 

Our study, discussing a new case of a rare Y;21 unbalanced translocation, highlighs the usefulness of the high-throughput molecular diagnostic methods for detection of subtle chromosomal rearrangements. To our knowledge, this is the first case in which a (Yp;21p) translocation has been ascertained using an array-CGH approach, thus reporting details of such a rearrangement at higher resolution. 
